# Salutogenic resource profiles and compassion fatigue among nurses: a latent profile analysis

**DOI:** 10.3389/fpubh.2026.1906172

**Published:** 2026-09-14

**Authors:** Shuangshuang Zhang, Meirong He, Weizeng Lou

**Affiliations:** Department of Nursing, Sir Run Run Shaw Hospital, Zhejiang University School of Medicine, Hangzhou, China

**Keywords:** compassion fatigue, latent profile analysis, mindfulness, nurses, perceived organizational support, sense of coherence

## Abstract

**Background:**

Compassion fatigue is a major occupational health concern among nurses and may be influenced by the availability of personal and organizational resources. Guided by the salutogenic model, this study aimed to identify latent profiles of nurses based on sense of coherence, perceived organizational support, and mindfulness, and to examine differences in compassion fatigue across these profiles.

**Methods:**

A total of 516 nurses were included in this study. Latent profile analysis was conducted using sense of coherence, perceived organizational support, and mindfulness as continuous profile indicators. Model selection was based on fit indices, entropy, profile size, parsimony, and theoretical interpretability. The Bolck–Croon–Hagenaars (BCH) method was used to compare compassion fatigue across the identified profiles while accounting for classification uncertainty. A manual BCH secondary model was further used to examine factors associated with compassion fatigue within each profile.

**Results:**

A two-profile solution was retained. The low-resource profile comprised 241 nurses (46.71%) and was characterised by below-average levels of sense of coherence, perceived organisational support, and mindfulness, whereas the high-resource profile comprised 275 nurses (53.29%) and showed above-average levels across all three indicators. Compassion fatigue was significantly higher in the low-resource profile than in the high-resource profile (estimated mean ± SE; 62.83 ± 1.70 vs. 30.68 ± 1.37; *p* < 0.001). In the profile-specific secondary analysis, working more than four night shifts per month was associated with higher compassion fatigue within the low-resource profile (B = 8.466, SE = 3.935, *p* = 0.031), whereas no demographic or work-related factors were significantly associated with compassion fatigue within the high-resource profile.

**Conclusion:**

Nurses exhibited two distinct salutogenic resource profiles that were associated with markedly different levels of compassion fatigue. These findings highlight the potential value of considering combined patterns of personal and organisational resources when identifying nurses with different levels of vulnerability to compassion fatigue. Prospective studies are needed to evaluate whether resource-support strategies tailored to these profiles can reduce compassion fatigue.

## 1 Introduction

Compassion fatigue is an important occupational health issue among nurses because nursing work requires continuous exposure to patients’ suffering, pain, deterioration, death, and emotional distress. Compassion fatigue within nursing practice has been conceptualized as a progressive state that may reduce nurses’ compassionate capacity and affect their emotional, psychological, and professional functioning, such as insomnia, exhaustion, depression, lower work satisfaction, loss of hope and failure to nurture, lack of spiritual awareness, poor judgment and discernment (1). A systematic review and meta-analysis reported that compassion fatigue is common among nurses and has important consequences for nurses, patients, and healthcare organizations, such as reduced ability to feel sympathy and empathy at work, strained nurse–patient relationship, increased medical disputes, increased costs for medical institutions (2). Therefore, identifying protective resources that may help nurses maintain well-being in demanding clinical environments is essential for compassion fatigue prevention.

The present study was guided by the salutogenic model of health. Salutogenesis, proposed by Antonovsky, shifts attention from the origins of disease to the origins of health and focuses on how individuals move toward the health end of the health ease/dis-ease continuum despite exposure to stressors (3). A central concept in this theory is sense of coherence, which reflects the extent to which individuals perceive life events as comprehensible, manageable, and meaningful (3). The salutogenic model also emphasizes generalized resistance resources, which are personal, social, material, cultural, and environmental resources that help individuals manage tension and cope effectively with stressors (3). From a salutogenic perspective, compassion fatigue may occur when nurses face repeated emotional and occupational stressors but lack sufficient resources to understand, manage, and find meaning in these experiences. Sense of coherence is particularly relevant in nursing because it may help nurses appraise clinical stressors as structured, manageable, and meaningful rather than overwhelming. A systematic review on sense of coherence in nurses concluded that sense of coherence is associated with work-related and individual factors and may serve as a health-promoting resource in nursing workplaces (4). Thus, sense of coherence can be regarded as a core personal salutogenic resource that may protect nurses from compassion fatigue.

In addition to sense of coherence, perceived organizational support may represent an organizational-level salutogenic resource. Perceived organizational support refers to employees’ global beliefs that the organization values their contributions and cares about their well-being (5). In clinical nursing settings, perceived organizational support may strengthen nurses’ sense of manageability by signaling that help, recognition, and work-related resources are available when demands become difficult. Recent nursing research has also linked perceived organisational support with compassion fatigue, suggesting that organisational support may be an important work-related resource for nurses’ psychological well-being (6).

Mindfulness may also be understood as a personal psychological resource within a salutogenic framework. Mindfulness refers to receptive attention to and awareness of present-moment experience, and it has been associated with psychological well-being (7). For nurses, mindfulness may support emotional regulation, reduce automatic stress reactions, and help maintain psychological balance when caring for patients in distress. Previous intervention research among oncology nurses found that a mindfulness-based intervention reduced burnout and compassion fatigue symptoms, supporting the potential protective role of mindfulness in nursing practice (8).

Although previous studies have examined factors associated with compassion fatigue, many have used variable-centered approaches that estimate average relationships across the whole sample. However, nurses may differ not only in the level of a single resource but also in the configuration of multiple resources. For example, some nurses may have high sense of coherence, strong organizational support, and high mindfulness, whereas others may show low resources across all three domains. Latent profile analysis is a person-centered approach that can identify unobserved subgroups of individuals who share similar patterns across continuous indicators (9). This approach is useful for examining heterogeneity within nursing populations and for identifying groups that may need different levels or types of intervention.

Therefore, this study aimed to identify latent profiles of nurses based on three salutogenic resources: sense of coherence, perceived organizational support, and mindfulness. The second aim was to compare compassion fatigue across the identified profiles while accounting for classification uncertainty. The third aim was to examine demographic and work-related factors associated with compassion fatigue within each latent profile. By identifying salutogenic resource profiles among nurses, this study may provide evidence for profile-specific strategies to prevent compassion fatigue and promote sustainable compassionate care.

## 2 Methods

### 2.1 Study design and participants

This multicentre cross-sectional study recruited registered nurses from 22 provinces or autonomous regions in mainland China using convenience and snowball sampling in September 2025. The research team first contacted nurses and managers through existing professional networks and personal contacts. These contact persons were informed about the study purpose, eligibility criteria and voluntary nature of participation, and were invited to distribute the survey link or QR code to eligible nurses in their hospitals and professional networks. Participants were also encouraged to forward the survey invitation to other eligible registered nurses, which helped extend recruitment across different provinces and regions. The participating nurses were recruited from tertiary, secondary and primary hospitals. Inclusion criteria were: (1) currently employed as a nurse in a clinical setting; (2) aged 18–60 years; (3) able to read and understand Chinese; and (4) willing to participate. Nurses with self-reported mental disorders or other conditions impairing their ability to complete the questionnaire were excluded. Data were collected via an online platform (Questionnaire Star, www.wjx.cn). Nurses accessed the anonymous questionnaire by scanning a QR code or clicking a survey link distributed by researchers. Electronic informed consent was obtained before questionnaire completion.

### 2.2 Sample size determination

Sample size was estimated using the formula for a single population proportion in a cross-sectional survey: n = Z^2^_α/2_p(1 − p)/d^2^. With a 95% confidence level (Z_α/2_ = 1.96), an expected proportion of 0.50 to obtain the maximum sample size, and a margin of error of 0.05, the minimum required sample size was 385. After allowing for a 20% non-response or invalid questionnaire rate, the required sample size was increased to approximately 482. A total of 516 nurses were finally included in the analysis, which exceeded the minimum requirement. In addition, there is no universally accepted closed-form sample size formula for latent profile analysis, sample size adequacy was evaluated in relation to model complexity, number and quality of indicators, class separation, and the size of the smallest profile. Previous methodological and simulation studies have shown that the ability to correctly identify latent profiles depends on sample size, class separation, indicator quality, number of indicators, and class size, and that model enumeration tends to be more stable when sample sizes are around 500 or larger under favorable conditions (10). Therefore, the final sample size of 516 nurses was considered acceptable for the planned LPA.

### 2.3 Measures

#### 2.3.1 Demographic and work-related characteristics

Demographic and work-related characteristics included age, gender, work experience (years), average monthly night shifts, annual income (¥), as specialist nurses, as manager, hospital level, mode of employment and professional title.

#### 2.3.2 Sense of coherence

Sense of coherence was measured using the 13-item sense of coherence scale (SOC-13) originally developed by Antonovsky with three dimensions, including comprehensibility, manageability and meaningfulness (3). The Chinese version of SOC-13 was translated and adapted by Bao et al.,with a good reliability of 0.76 (11). The items are rated with a 7-point Likert scale, and the total score ranges from 13 to 91 points, where higher values reflect stronger sense of coherence. The Cronbach’s αcoefficient of the scale in this study was 0.870.

#### 2.3.3 Compassion fatigue

Compassion fatigue was measured with the 13-item Compassion Fatigue Short Scale (CF-Short) originally developed by Adams et al. (12). The Chinese version was adapted and validated by Sun et al. (13). The instrument comprises two dimensions,including secondary traumatic stress with 5 items and burnout with 8 items. Items are rated on a 10-point Likert scale (1 = never to 10 = very frequently), producing a total score of 13–130 and higher scores indicate more severe compassion fatigue. Reported internal consistency for the total scale was 0.90. The Cronbach’s αcoefficient of the scale in this study was 0.954.

#### 2.3.4 Perceived organisational support

Perceived organisational support was measured using a 13-item Chinese adaptation derived from Eisenberger’s original scale (5). The scale comprises two dimensions, namely emotional support and instrumental support, with items rated on a five point Likert scale (1 = strongly disagree to 5 = strongly agree), yielding a total of 13–65 points. Higher totals reflect stronger perceived support (14). In the present study, the scale demonstrated high internal consistency, with a Cronbach’s α of 0.966 for the total scale. The Cronbach’s α coefficients were 0.961 for the emotional support subscale and 0.911 for the instrumental support subscale.

#### 2.3.5 Mindfulness

Mindfulness was assessed with the 15-item Mindful Attention Awareness Scale, developed by Brown and Ryan (7). The Chinese version scale was adapted and validated by Chen et al. (15). Participants rated each item on a 6-point Likert scale (1 = almost always to 6 = almost never). The total score ranges from 15 to 90 points with higher values reflecting greater mindful attention awareness. The Chinese version demonstrates good reliability, with Cronbach’s α of 0.89. The Cronbach’s αcoefficient of the scale in this study was 0.922.

### 2.4 Data quality control

Several procedures were used to reduce duplicate and low-quality responses. First, the online questionnaire was configured to allow only one submission from the same IP address. Second, all items were set as mandatory using the forced-response function, so questionnaires with missing responses could not be submitted. Third, before data analysis, all submitted questionnaires were screened for eligibility and response quality. Responses were considered invalid and excluded if obvious low-quality response patterns were identified, such as selecting the same response option for each scale. After data screening, all questionnaires were retained for analysis.

### 2.5 Statistical analysis

All statistical analyses were conducted using IBM SPSS Statistics and Mplus version 8.3. Continuous variables were summarised using means and standard deviations, whereas categorical variables were described using frequencies and percentages. All statistical tests were two-sided, and *p* < 0.05 was considered statistically significant.

Latent profile analysis was performed to identify unobserved subgroups of nurses based on sense of coherence, perceived organisational support, and mindfulness. These three variables were specified as continuous profile indicators and were standardised before model estimation because they were measured on different scales. Models with one to five profiles were estimated sequentially using robust maximum likelihood estimation (MLR), with 1,000 initial-stage random starts and 250 final-stage optimisations. Model selection was guided by the Akaike information criterion (AIC), Bayesian information criterion (BIC), sample-size-adjusted Bayesian information criterion (aBIC), entropy, the Lo–Mendell–Rubin likelihood ratio test (LMR), the adjusted Lo–Mendell–Rubin likelihood ratio test (aLMR), the bootstrap likelihood ratio test (BLRT), profile size, substantive interpretability, and model parsimony. Lower AIC, BIC, and aBIC values indicated better model fit, whereas entropy values closer to 1 indicated clearer classification. Entropy was not used as the sole criterion for model selection (10). Average posterior probabilities were additionally examined to assess classification quality. Profiles were labelled according to the pattern of standardised means across the three profile indicators, and the profile plot was created using the standardised class-specific means obtained from the final profile solution. For ease of interpretation, the final two-profile model was additionally estimated using the original, unstandardised indicator scores to obtain class-specific means and standard errors for sense of coherence, perceived organisational support, and mindfulness. Differences in these original-scale means between the two latent profiles were evaluated using model-constraint Wald tests. Because these three variables were used to define the latent profiles, these between-profile comparisons were interpreted descriptively rather than as independent confirmatory tests.

After the final two-profile solution had been retained, the Bolck–Croon–Hagenaars (BCH) method was used to compare compassion fatigue between the two profiles while accounting for classification uncertainty (16). Compassion fatigue was treated as a continuous distal outcome. Profile-specific means and standard errors were estimated, and the between-profile difference was evaluated using an overall Wald χ^2^ test.

A manual BCH secondary model was further estimated to examine factors associated with compassion fatigue within each latent profile. BCH weights were first saved from the final two-profile measurement model based on the standardised profile indicators. The saved BCH weights were subsequently specified as training variables in the secondary model, and compassion fatigue was regressed on demographic and work-related factors separately within each latent profile. The secondary model was estimated using MLR with no random starts (STARTS = 0). Age and work experience were entered as continuous covariates. Gender, average monthly night shifts, annual income, specialist nurse status, managerial role, hospital level, employment mode, and professional title were entered as binary-coded covariates. For continuous covariates, regression coefficients represented the expected change in compassion fatigue associated with a one-unit increase in the covariate. For binary-coded covariates, regression coefficients represented the adjusted mean difference in compassion fatigue for the group coded as 1 relative to the reference group coded as 0. Unstandardised regression coefficients (B), standard errors (SE), z values, and *p* values were reported.

## 3 Results

### 3.1 Characteristics of 516 nurses

A total of 516 nurses were included in this study (Table 1). The mean age of participants was 34.76 years (SD = 6.76), and the mean work experience was 12.85 years (SD = 7.60). Most participants were female (n = 457, 88.57%), while male nurses accounted for 11.43% (n = 59). Regarding work-related characteristics, 364 nurses (70.54%) reported four or fewer night shifts per month, whereas 152 nurses (29.46%) reported more than four night shifts per month. Most participants reported an annual income below ¥200,000 (n = 425, 82.36%). A total of 196 nurses (37.98%) were specialist nurses, and 112 nurses (21.71%) held managerial roles. Most participants worked in tertiary hospitals (n = 451, 87.40%) and held intermediate or senior title (n = 320, 62.02%).

**Table 1:** Characteristics of 516 nurses.

Variable	Mean±SD/Group	n	%
Age	34.76 ± 6.76	516	100.00%
Work experience (years)	12.85 ± 7.60	516	100.00%
Gender	Male	59	11.43%
	Female	457	88.57%
Night shifts	≤4	364	70.54%
	>4	152	29.46%
Annual income (¥)	≤200 k	425	82.36%
	>200 k	91	17.64%
As specialist nurses	Yes	196	37.98%
	No	320	62.02%
As manager	Yes	112	21.71%
	No	404	78.29%
Hospital level	Tertiary hospitals	451	87.40%
	Secondary hospitals or below	65	12.60%
Mode of employment	Staffing of government-affiliated institutions	210	40.70%
	Contractual employment	306	59.30%
Professional title	Junior title	196	37.98%
	Intermediate/senior title	320	62.02%

### 3.2 Latent profile model selection

As shown in Table 2, model fit indices generally improved as the number of profiles increased, with progressively lower AIC, BIC, and adjusted BIC values. However, the LMR and aLMR provided stronger support for the two-profile solution. The two-profile model showed significant LMR and adjusted LMR tests compared with the one-profile model (*p* < 0.001), whereas the corresponding tests for the three-profile model were not significant (LMR *p* = 0.0959; adjusted LMR *p* = 0.1025), indicating that the LMR and adjusted LMR tests did not provide evidence that the three-profile solution improved upon the two-profile solution. The LMR and adjusted LMR tests also remained non-significant for the four- and five-profile models. Although the BLRT remained significant across models and information criteria continued to decrease, the five-profile solution included a very small profile comprising only 0.4% of the sample. Considering statistical evidence, profile size, interpretability, and model parsimony, the two-profile solution was retained as the final model.

**Table 2:** Latent profile model selection.

Model	LL	AIC	BIC	aBIC	LMR *p*-value	aLMR *P*-value	BLRT *P*-value	Entropy	Profile proportion
1	−2196.517	4405.034	4430.510	4411.465	-	-	-	-	-
2	−2064.379	4148.758	4191.219	4159.477	<0.001	<0.001	<0.001	0.647	0.467/0.533
3	−2016.194	4060.388	4119.834	4075.395	0.0959	0.1025	<0.001	0.707	0.597/0.116/0.287
4	−2000.513	4037.027	4113.457	4056.322	0.3316	0.3393	<0.001	0.680	0.058/0.469/0.341/0.132
5	−1983.050	4010.099	4103.514	4033.681	0.1715	0.1786	<0.001	0.736	0.368/0.068/0.434/0.004/0.126

As shown in Table 3, the two-profile solution demonstrated acceptable classification quality. The average posterior probability for participants assigned to Class 1 was 0.890, whereas that for participants assigned to Class 2 was 0.902, supporting acceptable classification accuracy. Although the entropy value of 0.647 was moderate, the relatively high diagonal posterior probabilities suggested that most participants were assigned to their most likely profile with reasonable certainty.

**Table 3:** Average posterior probabilities for the two-profile solution.

Most likely profile	Class 1	Class 2
Class 1	0.890	0.110
Class 2	0.098	0.902

### 3.3 Profile characteristics

Table 4 shows the profile characteristics, while Figure 1 shows the standardised mean scores of profile indicators across the two latent profiles. The two-profile solution identified distinct patterns of psychological and organisational resources among nurses. Class 1, comprising 241 nurses (46.7%), showed below-average standardised scores for sense of coherence, perceived organisational support, and mindfulness, and was labelled the low-resource profile. Class 2, comprising 275 nurses (53.3%), showed above-average levels across all three indicators and was labelled the high-resource profile.

**Table 4:** Profile characteristics.

Profile	n (%)	Sense of coherence	Perceived organisational support	Mindfulness
Class 1: Low resource	241 (46.71%)	−0.682	−0.584	−0.682
Class 2: High resource	275 (53.29%)	0.599	0.512	0.599

**Figure 1:**
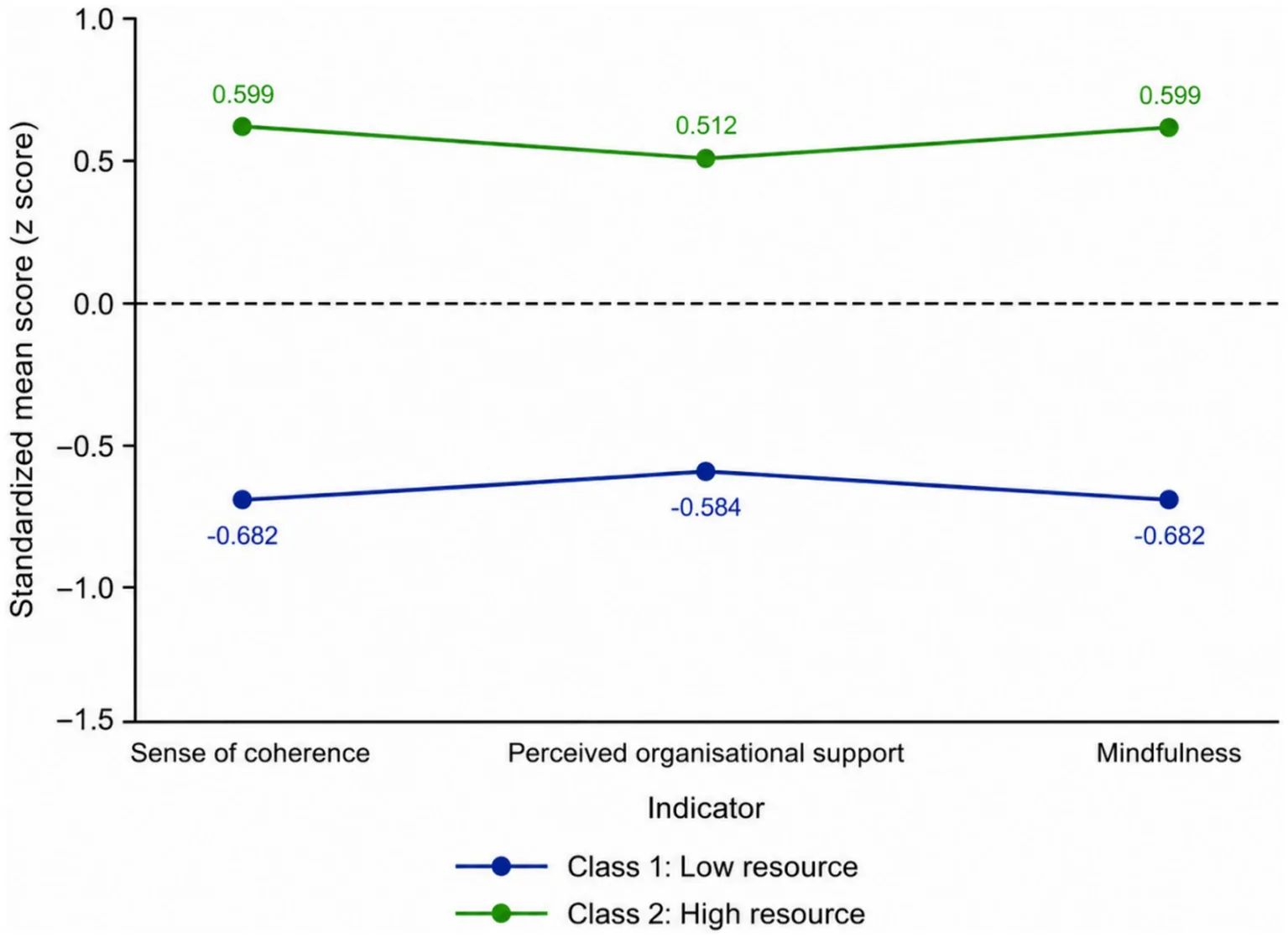
Standardised mean scores of profile indicators across the two latent profiles. Values are standardised scores; zero represents the sample mean.

### 3.4 Mean and standard error (SE) of study variables across the identified latent profiles

Table 5 presents the mean and standard error (SE) of the study variables for the overall sample and the two latent profiles. In the overall sample, the mean ± SE values were 59.77 ± 0.55 for sense of coherence, 46.05 ± 0.43 for perceived organisational support, 65.96 ± 0.57 for mindfulness, and 45.71 ± 1.08 for compassion fatigue. The two latent profiles showed clearly distinct patterns across the three resource indicators. Compared with the high-resource profile (Class 2), the low-resource profile (Class 1) had lower estimated mean scores for sense of coherence (51.30 ± 1.17 vs. 67.20 ± 1.17), perceived organisational support (40.37 ± 1.14 vs. 51.04 ± 0.64), and mindfulness (57.07 ± 1.18 vs. 73.77 ± 1.34) (*p* < 0.001). In contrast, compassion fatigue was substantially higher in the low-resource profile than in the high-resource profile (62.83 ± 1.70 vs. 30.68 ± 1.37, *p* < 0.001). Overall, the low-resource profile was characterised by lower salutogenic resources and markedly higher compassion fatigue, whereas the high-resource profile showed the opposite pattern.

**Table 5:** Means and SE of study variables across the identified latent profiles (N = 516).

Variables	Overall, Mean ± SE	Class 1, Mean ± SE	Class 2, Mean ± SE	Class 1 vs. Class 2, *P*-value
Sense of coherence	59.77 ± 0.55	51.30 ± 1.17	67.20 ± 1.17	<0.001*
Perceived organizational support	46.05 ± 0.43	40.37 ± 1.14	51.04 ± 0.64	<0.001*
Mindfulness	65.96 ± 0.57	57.07 ± 1.18	73.77 ± 1.34	<0.001*
Compassion fatigue	45.71 ± 1.08	62.83 ± 1.70	30.68 ± 1.37	<0.001*

Between-profile *p* values for sense of coherence, perceived organisational support, and mindfulness were based on Wald tests of class-specific mean differences. The *p* value for compassion fatigue was obtained from the BCH overall Wald χ^2^ test. *p < 0.001.

### 3.5 Factors associated with compassion fatigue within the two latent profiles

As shown in Table 6, among nurses in the low-resource profile, more frequent night shifts were significantly associated with higher compassion fatigue after adjustment for the other demographic and work-related variables (B = 8.466, SE = 3.935, z = 2.151, *p* = 0.031). Specifically, nurses working more than four night shifts per month had compassion fatigue scores approximately 8.47 points higher than those working four or fewer night shifts per month. None of the other examined variables were significantly associated with compassion fatigue in the low-resource profile (all *p* > 0.05). In the high-resource profile, none of the demographic or work-related variables showed a statistically significant association with compassion fatigue (all *p* > 0.05).

**Table 6:** Factors associated with compassion fatigue within the two latent profiles.

	Class 1: Low-resource profile				Class 2: High-resource profile			
Variable	B	SE	z	*p* value	B	SE	*z*	*p* value
Age	−0.285	1.107	−0.257	0.797	−0.436	0.898	−0.486	0.627
Gender: male (ref:female)	6.286	5.069	1.240	0.215	2.079	7.025	0.295	0.767
Working experience	−0.251	0.965	−0.260	0.795	0.629	0.772	0.814	0.415
Night shifts: >4/month (ref:≤4/month)	8.466	3.935	2.151	0.031*	−2.702	3.694	−0.731	0.464
Annual income: >200 k/year (ref:≤200 k/year)	−0.927	4.436	−0.209	0.834	−1.225	3.475	−0.352	0.724
Specialist nurse: yes (ref: no)	−1.384	3.436	−0.403	0.687	3.527	3.431	1.028	0.304
Manager: yes (ref: no)	4.372	4.461	0.980	0.327	−5.705	3.699	−1.543	0.123
Hospital level: Tertiary hospital (ref: Secondary hospitals or below)	7.823	4.468	1.751	0.080	7.214	5.812	1.241	0.214
Employment mode: Staffing of government-affiliated institutions (ref: Contractual employment)	−0.365	3.797	−0.096	0.923	2.778	3.546	0.783	0.433
Professional title: Junior title (ref: Intermediate/senior title)	9.137	5.367	1.703	0.089	−8.184	4.918	−1.664	0.096

B = unstandardized regression coefficient; SE = standard error; z = B/SE. Class 1 represents the low-resource profile and Class 2 represents the high-resource profile. Regression estimates were obtained using the manual BCH approach to account for classification uncertainty. Reference categories are shown in parentheses. *p < 0.05. Bold *p* values indicate statistical significance at *p* < 0.05.

## 4 Discussion

This study used a person-centred latent profile approach to examine heterogeneity in salutogenic resources among nurses. A two-profile solution was retained, comprising a low-resource profile (46.7%) and a high-resource profile (53.3%). Nurses in the low-resource profile showed consistently below-average levels of sense of coherence, perceived organisational support, and mindfulness, whereas those in the high-resource profile showed above-average levels across all three indicators. Compassion fatigue was substantially higher in the low-resource profile than in the high-resource profile. These findings suggest that combinations of personal and organisational salutogenic resources are associated with meaningful differences in compassion fatigue among nurses. Notably, the two profiles differed primarily in the overall level of the three salutogenic resources rather than showing markedly different configurations across indicators. In other words, sense of coherence, perceived organisational support, and mindfulness tended to vary in the same direction within each profile. Thus, the present profiles may be interpreted primarily as representing lower versus higher overall salutogenic resource availability rather than qualitatively distinct resource configurations.

The findings are consistent with the salutogenic model of health. Salutogenesis emphasizes that health is not simply the absence of disease but a dynamic movement toward the health end of a continuum, supported by sense of coherence and resistance resources (17). In the present study, nurses in the low-resource profile had simultaneously low sense of coherence, low perceived organizational support, and low mindfulness. This pattern may indicate limited capacity to understand clinical stressors, access or perceive available support, regulate emotional responses, and maintain meaning in demanding care situations. According to the salutogenic model, insufficient resistance resources may weaken individuals’ ability to manage tension and maintain health under stress (18).

The high-resource profile showed lower compassion fatigue, suggesting that higher combined levels of these resources may be associated with lower vulnerability to compassion fatigue. A strong sense of coherence may help nurses perceive complex clinical situations as more comprehensible, manageable, and meaningful (3). Perceived organizational support may strengthen nurses’ belief that the organization values their contribution and will provide assistance when work demands exceed individual capacity (5). Mindfulness may further help nurses remain aware of present-moment experiences and regulate distressing emotions without becoming overwhelmed by them (7). Together, higher levels of these resources may be associated with greater availability of internal and external coping resources and, consequently, lower vulnerability to compassion fatigue.

The findings also extend previous nursing research by showing that compassion fatigue is associated with patterns of resources rather than only isolated predictors. Previous evidence indicates that SOC may be a health-promoting resource among nurses and is associated with work-related outcomes such as distress, burnout, and job satisfaction (4). Other studies have shown that perceived organizational support is related to compassion fatigue among nurses, including Chinese nursing samples (19). Mindfulness-based interventions have also been shown to reduce burnout and compassion fatigue symptoms in nurses (8). The present study integrates these lines of evidence by demonstrating that these resources cluster into meaningful profiles that correspond to different levels of compassion fatigue.

The profile-specific secondary analyses explored factors associated with compassion fatigue within each resource profile. In the profile-specific secondary analysis, working more than four night shifts per month was associated with higher compassion fatigue within the low-resource profile after adjustment for other demographic and work-related factors. This finding is consistent with previous evidence linking night-shift work to compassion fatigue among nurses. Fu et al. reported that the number of night shifts per week was significantly associated with compassion fatigue among operating room nurses in China, with more frequent night shifts corresponding to higher levels of compassion fatigue (20). Similarly, Al-Hammouri et al. found that nurses working rotating shifts had poorer sleep quality and higher levels of burnout and secondary traumatic stress than nurses working fixed shifts (21). Frequent night-shift work may disrupt normal work–rest patterns, impair recovery, and increase cumulative physical and psychological demands, which may contribute to greater vulnerability to compassion fatigue. In the high-resource profile, none of the demographic or work-related variables were significantly associated with compassion fatigue. However, the absence of statistically significant associations in this profile should not be interpreted as evidence that high salutogenic resources buffer the effects of demographic or occupational factors, because differences in regression coefficients between the two profiles were not formally tested. Further studies are needed to examine whether salutogenic resources modify associations between occupational exposures and compassion fatigue.

### 4.1 Implications

The present findings highlight the potential value of considering nurses’ personal and organisational resources jointly rather than focusing only on compassion fatigue scores or isolated resource indicators. A person-centered approach may help identify subgroups with different overall patterns or levels of salutogenic resources and may provide a basis for more targeted prevention and intervention strategies (22, 23). In particular, nurses characterised by relatively low sense of coherence, perceived organisational support, and mindfulness may warrant greater attention because this profile was associated with substantially higher compassion fatigue, which may help inform the prospective development and evaluation of resource-oriented interventions. Potential strategies for nurses with relatively low resources may include psychological support, peer support, mentorship, supervisor support, mindfulness-based programmes, and opportunities to strengthen coping and professional meaning. Previous reviews and intervention studies suggest that behavioural, psychological, mindfulness-based, and multicomponent interventions may help reduce compassion fatigue, burnout, and secondary traumatic stress among nurses (8, 24, 25).

The findings also underscore the importance of organisational conditions. In the low-resource profile, working more than four night shifts per month was associated with higher compassion fatigue, suggesting that work scheduling may be an important consideration when supporting nurses with relatively limited salutogenic resources. Healthcare organisations may therefore consider strategies such as fair scheduling, adequate staffing, reasonable night-shift allocation, supportive leadership, recognition of nurses’ contributions, access to psychological support, and opportunities for participation in decision-making. Importantly, compassion fatigue prevention should not place responsibility solely on individual nurses. Previous evidence indicates that workplace support, job control, workload, reward, and supervisor support are associated with burnout-related outcomes, supporting the importance of organisational-level approaches (26). From a salutogenic perspective, combining individual resource-building with supportive organisational conditions may be more appropriate than relying on individual-level interventions alone. Nevertheless, longitudinal and intervention studies are needed to determine whether modifying these resources or working conditions leads to reductions in compassion fatigue.

### 4.2 Limitations

This study has several limitations. First, the cross-sectional design prevents causal inference. Although lower salutogenic resources were associated with higher compassion fatigue, the temporal direction of these relationships cannot be established, and reverse or bidirectional associations are possible. Longitudinal studies are therefore needed to examine temporal relationships among salutogenic resources and compassion fatigue. Second, although participants were recruited from 22 provinces or autonomous regions in mainland China, convenience and snowball sampling were used, and 87.40% of participants worked in tertiary hospitals. Therefore, the findings may not generalise to nurses working in primary or secondary hospitals or to the broader Chinese nursing workforce. Third, all variables were assessed using self-report questionnaires, which may introduce common-method and social-desirability bias. Fourth, the latent profiles were derived from only three salutogenic resource indicators, and the two-profile solution primarily reflected overall differences in resource levels. Classification quality was acceptable but moderate, with an entropy value of 0.647. Future studies should replicate the solution in independent and more diverse samples and include a broader range of personal, interpersonal, and organisational resources.

## 5 Conclusion

This study identified two salutogenic resource profiles among nurses: a low-resource profile and a high-resource profile. The low-resource profile showed substantially higher compassion fatigue than the high-resource profile, indicating that combined patterns of sense of coherence, perceived organisational support, and mindfulness are associated with compassion fatigue. Frequent night-shift work was additionally associated with higher compassion fatigue within the low-resource profile, although this secondary finding requires cautious interpretation and replication. These findings may inform the development and prospective evaluation of resource-oriented strategies to support nurses’ occupational well-being. Longitudinal and intervention studies are needed before conclusions regarding causality or intervention effectiveness can be drawn.

## Data Availability

The raw data supporting the conclusions of this article are available from the first author upon reasonable request.
